# Factors Associated With Persistence of Plasma HIV-1 RNA During Long-term Continuously Suppressive Firstline Antiretroviral Therapy

**DOI:** 10.1093/ofid/ofy032

**Published:** 2018-02-03

**Authors:** Alessandra Ruggiero, Alessandro Cozzi-Lepri, Apostolos Beloukas, Douglas Richman, Saye Khoo, Andrew Phillips, Anna Maria Geretti, ERAS Study Group

**Affiliations:** 1 Institute of Infection and Global Health, University of Liverpool, Liverpool, United Kingdom; 2 Department of Infection and Population Health, University College London, London, United Kingdom; 3 VA San Diego Healthcare System and Center for AIDS Research, University of California San Diego, La Jolla, California; 4 Institute of Translational Medicine, University of Liverpool, Liverpool, United Kingdom

**Keywords:** 2-LTR circular DNA, activation, drug concentration, sCD27, viral load

## Abstract

**Background:**

Persistence of plasma HIV-1 RNA during seemingly effective antiretroviral thereapy (ART) is incompletely understood. Using an ultrasensitive assay, this cross-sectional study investigated residual plasma HIV-1 RNA in subjects maintained on firstline ART with continuous viral load suppression <50 copies/mL for ≤15 years without recognized viral load blips or treatment interruptions and explored its relationship with the duration of suppressive ART, efavirenz concentrations in plasma, 2-LTR circular HIV-1 DNA (2-LTRc DNA) in peripheral blood mononuclear cells, and cellular (CD4 plus CD26/CD38/CD69; CD8 plus CD38/HLA-DR/DP/DQ) and soluble (sCD14, sCD27, sCD30, IL-6) markers of immune activation in peripheral blood.

**Methods:**

Residual plasma HIV-1 RNA, total HIV-1 DNA and 2-LTRc DNA were quantified by real-time and digital droplet PCR. Cellular (CD4 plus CD26/CD38/CD69; CD8 plus CD38/HLA-DR/DP/DQ) and soluble (sCD14, sCD27, sCD30, IL-6) markers of immune activation were measured by flow cytometry and ELISA.

**Results:**

Residual plasma HIV-1 RNA and 2-LTRc DNA were detected in 52/104 (50%) and 24/104 (23%) subjects, respectively. Among subjects with detectable HIV-1 RNA, 50/52 showed levels ≤11 copies/mL. In adjusted analyses, HIV-1 RNA levels were 0.37 log_10_ copies/mL higher with each log_10_ U/mL increase in sCD27 (95% confidence interval, 0.01–0.73; *P* = .02). No significant association was found between residual plasma HIV-1 RNA and other explored parameters.

**Conclusions:**

These findings point to an ongoing relationship between plasma HIV-1 RNA and selected markers of immune activation during continuously suppressive ART. The novel direct association with levels of sCD27 warrants further investigation.

Maintaining plasma HIV-1 RNA suppression below the limit of detection of commercial viral load assays defines clinical success of antiretroviral therapy (ART) [1, 2]. Ultrasensitive assays can detect traces of HIV-1 RNA in the plasma of many successfully treated patients [3], but the source remains controversial [4, 5]. On the one hand, it is proposed that detection of residual plasma HIV-1 RNA reflects short-lived bursts of virus production from latently infected memory CD4 T cells, but with ongoing virus replication and genetic evolution inhibited by ART [6]. An alternative model derives from evidence of ongoing virus replication in compartments where drug penetration or activity is suboptimal [7–10]. While the 2 models are not mutually exclusive, published studies have often included patients with heterogeneous treatment histories and varying definitions of suppression, which may in part explain conflicting findings [11–13].

Identifying the determinants of HIV-1 RNA persistence during seemingly effective ART is central to our understanding of how the virus may be eradicated [14, 15]. Such studies must also consider the interplay between virus replication and host immunity, and the role of chronic immune activation and inflammation in disease pathogenesis [16–18]. Effective ART reduces but does not always normalize inflammatory markers, raising the possibility that residual immune activation may both sustain and be sustained by residual virus replication [18]. Available data are not conclusive, however, and they rather point to virologic and immunologic events that take place before the initiation of ART as key determinants of the HIV-1 reservoirs and immune activation that can persist despite ART [19].

We previously reported that treated patients with plasma HIV-1 RNA detected at levels ranging between approximately 10 and 49 copies/mL by a commercial viral load assay were more likely to experience viral load rebound above 50 copies/mL and above 400 copies/mL during 12 months of follow-up when compared with treated patients with undetectable HIV-1 RNA [20]. The aim of this study was to take the converse approach and assess a population that experienced continuous viral load suppression <50 copies/mL for up to 15 years of firstline ART and use ultrasensitive testing to detect HIV-1 RNA below the 10-copies/mL cutoff. The cohort was selected according to precise inclusion criteria, and residual plasma HIV-1 RNA was investigated cross-sectionally in relation to the duration of suppressive ART, plasma concentrations of efavirenz, 2-LTRc DNA in peripheral blood mononuclear cells (PBMCs), and cellular and soluble markers of immune activation in peripheral blood.

## METHODS

### Study Population

In this cross-sectional study, eligible patients had started firstline ART with 2 nucleoside/nucleotide reverse transcriptase inhibitors (NRTIs) plus 1 non-nucleoside reverse transcriptase inhibitor (NNRTI), had achieved viral load suppression <50 copies/mL within 6 months of starting ART, and during subsequent follow-up had remained on the initial NNRTI, undergone ≥2 viral load measurements per year, and maintained continuous viral load suppression <50 copies/mL without any recorded viral load elevation >50 copies/mL or treatment interruption. To ensure balance in terms of duration of suppressive ART, recruitment was stratified to span from 1 year of treatment to 10 years or longer. Changes of the initial NNRTI were not allowed. Changes of the initial NRTIs were allowed to reflect evolving practice, provided they did not coincide with a viral load >50 copies/mL or a treatment interruption. The study took place at multiple centers across the United Kingdom. It was approved by the National Research Ethics Service (London-Dulwich) and included in the National Institute for Health Research Clinical Research Network Portfolio. Patients provided written informed consent.

### Residual Plasma HIV-1 RNA

Plasma was separated from venous blood in EDTA within 2 hours of collection and stored at −80°. HIV-1 RNA was quantified using a modified version of the RealTime HIV-1 assay (Abbott, Maidenhead, UK). Validation studies previously showed that the assay 50% and 95% detection rates were 1 and 3 HIV-1 RNA copies/mL, respectively, with high reproducibility across diverse HIV-1 subtypes [10, 20, 21].

### Total HIV-1 DNA and 2-LTRc DNA

PBMCs were isolated by Ficoll-Hypaque gradient centrifugation and cryopreserved. Total HIV-1 DNA was quantified by real-time polymerase chain reaction (PCR) as described [22]; the assay 50% and 95% detection rates were 20 and 40 HIV-1 DNA copies/10^6^ PBMC, respectively. 2-LTRc DNA was measured by droplet digital PCR as described [23]; the assay’s lower limit of detection was a median (interquartile range [IQR]) of 5 (4–6) copies/10^6^ PBMC. 

### Cellular and Soluble Markers of Immune Activation

Flow cytometry was performed at the Immunology Service of the Royal Free Hospital in London using freshly collected blood. Following centrifugation, cell pellets were lysed by 10-minute incubation at room temperature in 1× Hoffman’s lysis buffer and incubated with FITC-, Cy5- or PE-labeled antibodies targeting CD4 plus CD26, CD38, or CD69, and CD8 plus CD38 or HLA-DR/DP/DQ (Becton Dickinson, Swindon, UK). Labeled cells were incubated in 1% paraformaldehyde for 30 minutes prior to acquisition with BD FACSCalibur and analysis by CellQuest. Soluble markers were measured in frozen plasma by commercial Elisa assays (EIAs) for soluble CD14 (sCD14, R&D system, London, UK; assay sensitivity ≥125 × 10^−6^ µg/mL), soluble CD27 (sCD27, EBiosciences; ThermoFisher, Loughborough, UK; assay sensitivity ≥0.2 U/mL), soluble CD30 (sCD30, EBiosciences, UK; assay sensitivity ≥0.3 ng/mL), and IL6 (EBiosciences, UK, assay sensitivity ≥0.9 pg/mL).

### Plasma Drug Concentrations

Efavirenz (EFV) and nevirapine (NVP) concentrations were measured in untimed plasma samples using high-performance liquid chromatography–tandem mass spectrometry, as previously described [10]. The time of sample collection relative to last dosing was recorded.

### Analysis

Duration of suppressive ART was defined as the length of time following the first viral load measurement <50 copies/mL, which in all subjects was recorded within 6 months of starting ART. When analyzing residual plasma HIV-1 RNA and 2-LTRc DNA as continuous variables, undetectable values were replaced with the midpoint value between 0 and the lower limit of detection of the assays. EFV and NVP concentrations were categorized as above or below the recommended minimal effective concentration (MEC) for wild-type virus: 1000 ng/mL for EFV [24] and 3400 ng/mL for NVP [25]. Participants’ characteristics were compared by analysis of variance (ANOVA), the Kruskal-Wallis test, and the Fisher exact test for continuous and categorical variables, as appropriate. Data distribution was assessed through descriptive scatter plots. The correlation between EFV concentrations and levels of residual plasma HIV-1 RNA and 2-LTRc DNA was measured by Spearman’s rank test. Given that all but 2 HIV-1 RNA values were ≤11 copies/mL, in order to reduce heterogeneity, the main statistical models excluded 2 subjects with residual plasma HIV-1 RNA >11 copies/mL (referred to as outliers). This was based on our previous data indicating that patients on stable ART with a viral load above approximately 10 copies/mL are a distinct population at risk of viral load rebound [20]. Three main models were run. The mean difference (with 95% confidence interval [CI]) in residual plasma HIV-1 RNA, total HIV-1 DNA, 2-LTRc DNA, and markers of immune activation according to duration of suppressive ART (normalized over 1 year) was analyzed by univariable linear regression analysis after log-transformation of the variables. Factors associated with levels of residual plasma HIV-1 RNA or 2-LTRc DNA were investigated by univariable and multivariable linear regression analysis. Variables associated (*P* ≤ .20) with the outcome of interest in the univariable analysis were included in the multivariable models, with the following modifications: (a) when modeling 2-LTRc DNA, total HIV-1 DNA was excluded due to high colinearity (*P* < .0001); (b) age and duration of ART were excluded in favor of duration of suppressive ART. Separate models explored: (a) the effect of forcing the nadir CD4 cell count and pre-ART viral load into the multivariable analyses and (b) the association between residual plasma HIV-1 RNA or 2-LTRc DNA and plasma concentrations of EFV (as a continuous variable) in EFV recipients, after adjustment for the time of sampling in relation to the last EFV dose. IBM SPSS v. 22.0 (Armonk, NY, USA) and SAS v9.4 (Cary, NC, USA) were used for the analyses.

## RESULTS

### Study Population

The population comprised 104 patients that at the time of recruitment had experienced continuous viral load suppression <50 copies/mL for a median (IQR) of 5 (3–8) years while receiving firstline ART with 2 NRTIs plus either EFV (n = 86, 83%) or NVP (Table 1). Reflecting evolving guidelines about when to start ART and which antiretrovirals to use, patients with longer duration of suppressive ART had a lower nadir CD4 cell count and a higher pre-ART viral load, were more often receiving NVP, and were more likely to have experienced changes of the NRTI backbone after first starting ART: 48/104 (48%) patients changed ≥1 NRTIs prior to recruitment, with a median (IQR) of 1 (1–2) NRTI change per subject. At recruitment, NRTI backbones comprised tenofovir disoproxil fumarate (78/104, 75%), abacavir (22/104, 21%) or zidovudine (4/104, 4%), plus emtricitabine or lamivudine.

**Table 1.** Characteristics of the Study Population Overall and by Duration of Suppressive ART (n = 104)^a^

		Total (n = 104)	Duration of Suppressive ART, y		
			<4 (n = 34)	4–7 (n = 35)	>7 (n = 35)
Duration of suppressive ART, median (IQR), y		5 (3–8)	2 (2–3)	5 (5–6)	9 (8–10)
Age, median (IQR), y		47 (40–53)	44 (36–49)	49 (39–53)	48 (43–55)
Male, n (%)		81 (78)	29 (85)	29 (83)	23 (66)
Ethnicity, n (%)	White	59 (57)	20 (59)	22 (63)	17 (49)
	Black	41 (39)	13 (38)	11 (31)	17 (49)
	Asian	4 (4)	1 (3)	2 (6)	1 (3)
Pre-ART viral load, median (IQR), log_10_ cps/mL		4.9 (4.6–5.3)	4.7 (4.1–5.1)	4.9 (4.6–5.3)	5.1 (4.8–5.6)
Nadir CD4 count, median (IQR), cells/mm^3^		201 (110–270)	279 (216–365)	166 (126–238)	118 (79–215)
EFV use, n (%)		86 (83)	30 (88)	32 (89)	24 (69)
NNRTI concentration, median (IQR), ng/mL	EFV	1531 (846–2235)	1492 (735–2130)	1496 (866–2154)	1966 (855–2626)
	NVP	5889 (3096–7419)	4545 (2798–6147)	2734 (2539–5743)	6311 (4390–7603)
NNRTI concentration above target MEC^b^, n (%)		71 (66)	22 (65)	24 (69)	25 (71)
Changed initial NRTIs, n (%)		48 (46)	4 (12)	13 (37)	31 (89)
Detectable residual plasma HIV-1 RNA, n (%)		52 (50)	21 (62)	18 (51)	13 (37)
Residual plasma HIV-1 RNA, median (IQR),^c^ cps/mL		2 (2–4)	2 (2–4)	2 (2–4)	2 (2–2)
Detectable 2-LTRc DNA, n (%)		24 (23)	12 (35)	7 (20)	5 (14)
2-LTRc DNA, median (IQR),^d^ cps/10^6^ PBMC		3 (3–3)	3 (3–7)	3 (3–3)	3 (3–3)
Total HIV-1 DNA, median (IQR), log_10_ cps/10^6^ PBMC		2.5 (2.1–2.8)	2.6 (2.3–3.0)	2.4 (1.7–2.8)	2.5 (2.1–2.8)
CD4 count, median (IQR), cells/mm^3^		581 (474–722)	548 (427–629)	580 (509–720)	632 (503–796)
CD8 count, median (IQR), cells/mm^3^		798 (656–1047)	808 (596–1060)	818 (685–1024)	679 (496–929)
CD4^+^ CD38^+^, median (IQR), %		24 (16–34)	32 (23–36)	21 (15–31)	22 (15–28)
CD4^+^CD26^+^, median (IQR), %		58 (46–64)	56 (47–67)	59 (49–63)	58 (44–64)
CD4^+^CD69^+^, median (IQR), %		1 (1–2)	2 (1–3)	1 (1–2)	2 (1–2)
CD8^+^CD38^+^, median (IQR), %		5 (3–7)	6 (4–8)	4 (3–6)	4 (3–6)
CD8^+^HLA-DR/DP/DQ^+^, median (IQR), %		35 (24–42)	37 (27–47)	35 (26–40)	30 (20–39)
sCD14, median (IQR), µg/mL		2.2 (1.8–2.5)	2.2 (1.8–2.4)	2.0 (1.8–2.5)	2.3 (1.9–2.5)
sCD27, median (IQR), Ug/mL		62 (49–76)	64 (52–80)	61 (54–76)	60 (46–71)
sCD30, median (IQR), ng/mL		11 (9–15)	11 (9–15)	12(10–17)	11 (9–13)
IL6 median (IQR), pg/mL		0.8 (0.5–2.1)	0.5 (0.5–1.2)	1.1 (0.5–2.3)	0.8 (0.5–2.1)

Abbreviations: 2-LTRc DNA, 2-LTR circular HIV-1 DNA; ART, antiretroviral therapy; cps, copies; EFV, efavirenz; IQR, interquartile range; MEC, minimum effective concentration; NNRTI, non-nucleoside reverse transcriptase inhibitor; NRTI, nucleoside/nucleotide reverse transcriptase inhibitor; NVP, nevirapine; PBMC, peripheral blood mononuclear cell.

^a^Defined as the length of time between the first viral load <50 copies/mL measured after starting ART and the date of recruitment.

^b^Minimum effective concentration recommended for wild-type virus (1000 ng/mL for EFV and 3400 ng/mL for NVP).

^c^Samples with undetectable HIV-1 RNA were assigned an arbitrary value of 1.5 copies/mL.

^d^Samples with undetectable 2-LTRc DNA were assigned an arbitrary value of 2.5 copies/10^6^ PBMC.

**Figure 1.**
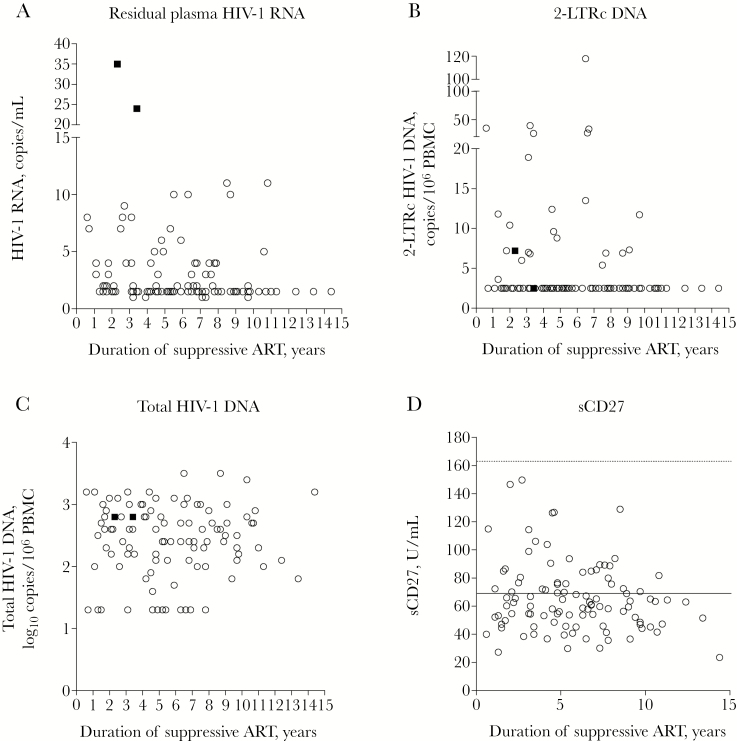
Virologic and immunologic parameters in patients with up to 15 years of consistently suppressive antiretroviral therapy (ART; n = 104), comprising (A) residual plasma HIV-1 RNA (copies/mL), (B) 2-LTR circular HIV-1 DNA (2-LTRc DNA; copies per 10^6^ peripheral blood mononuclear cell [PBMC]), (C) total HIV-1 DNA (log_10_ copies per 10^6^ PBMC), and (D) sCD27 (U/mL) in relation to the duration of suppressive ART (years). In (D), the dotted line indicates the mean sCD27 levels reported in HIV-negative volunteers in published studies (164 U/mL) [25]; the solid lines indicate the median values measured in the whole study population. Each circle represents 1 participant; the 2 solid squares represent the 2 patients with residual plasma HIV-1 RNA >11 copies/mL.

Residual plasma HIV-1 RNA was detected in 52/104 (50%) patients (Table 1). Levels ranged between 1 and 35 copies/mL and were ≤11 copies/mL in 50/52 (96%) patients; levels in the 2 outliers were 35 and 24 copies/mL after 2 and 3 years of suppressive ART, respectively (Figure 1). 2-LTRc HIV-1 DNA was detected in 24/104 (23%) patients with median levels of 3 copies/10^6^ PBMC that ranged between 4 and 119 copies/10^6^ PBMC (Figure 1). Cellular (Supplementary Figure 1) and soluble (Figure 1; Supplementary Figure 2) markers of immune activation were detected in all patients. Levels of soluble markers were generally not dissimilar from those of HIV-negative volunteers reported in published studies [26–29]. When analyzing subjects whose levels of soluble markers fell above the upper quartile of the whole study population, a few patients presented concomitant elevations, but with little evidence of consistent patterns. Overall 5/104 (5%) patients showed concomitant elevations of >2 soluble markers, comprising sCD14 + sCD27 + sCD30 (3/104, 3%); CD27 + sCD30 + IL-6 (1/104, 1%); and sCD14 + sCD27 + sCD30 + IL-6 (1/104, 1%) (Supplementary Table 1). CD8 cells counts and frequency of CD8^+^HLA-DR/DP/DQ^+^ cells were often, albeit not always, increased in these patients, but there were no apparent patterns in other parameters.

### Virologic and Immunologic Parameters in Relation to the Duration of Suppressive ART

Differences in virologic and immunologic parameters according to the duration of suppressive ART, analyzed after exclusion of the 2 outliers, are shown in Table 2. Patients with longer duration of suppressive ART had higher CD4 counts (+19 cells/mm^3^/y) and lower expression of activation markers on CD4 cells (CD38) and CD8 cells (CD38, HLA-DR/DP/DQ). Levels of sCD14, sCD30, and IL-6 did not differ by duration of suppressive ART, whereas levels of sCD27 were significantly lower with longer duration of suppressive ART. A trend was observed for lower levels of 2-LTRc DNA with longer duration of suppressive ART. There was less evidence of a difference in residual plasma HIV-1 RNA, and no significant difference was seen in total HIV-1 DNA. A linear regression analysis confirmed an inverse association between 2-LTRc DNA and duration of suppressive ART, with an adjusted mean difference of 0.03 log_10_ copies/10^6^ PBMC per year longer (95% CI, –0.05 to –0.00; *P* = .02) (Supplementary Table 2). Forcing the nadir CD4 cell count and pre-ART viral load into the multivariable model did not change the findings: 2-LTRc DNA levels were 0.03 log_10_ copies/10^6^ PBMC lower per year of suppressive ART (95% CI, –0.05 to –0.00; *P* = .02; not shown).

**Table 2.** Univariable Analysis of the Mean Difference in Virologic and Immunologic Parameters per 1 Year of Suppressive ART (n = 102)^a^

	Mean Difference^b^	95% CI	*P*
Residual plasma HIV-1 RNA, cps/mL	–0.013	–0.030 to 0.004	.12
2-LTRc DNA, cps/10^6^ PBMC	–0.018	–0.039 to 0.003	.09
Total HIV-1 DNA, cps/10^6^ PBMC	0.004	–0.033 to 0.042	.82
CD4 count, cells/mm^3^	0.015	0.004 to 0.026	.01
CD8 count, cells/mm^3^	–0.004	–0.018 to –0.009	.52
CD4^+^CD38^+^, %	–0.022	–0.034 to –0.009	.001
CD4^+^CD26^+^, %	–0.003	–0.012 to 0.005	.44
CD4^+^CD69^+^, %	–0.008	–0.021 to 0.005	.21
CD8^+^CD38^+^, %	–0.014	–0.032 to 0.004	.05
CD8^+^HLA-DR/DP/DQ^+^, %	–0.014	–0.027 to 0.001	.04
sCD14, µg/mL	0.002	–0.004 to 0.009	.50
sCD27, U/mL	–0.010	–0.019 to 0.000	.05
sCD30, ng/mL	–0.005	–0.019 to 0.009	.44
IL-6, pg/mL	0.003	–0.015 to 0.022	.72

Abbreviations: 2-LTRc DNA, 2-LTR circular HIV-1 DNA; CI, confidence interval; PBMC, peripheral blood mononuclear cell.

^a^The analysis excluded the 2 outliers with residual plasma HIV-1 RNA >11 copies/mL.

^b^Variables were log-transformed prior to the analysis.

### Factors Associated With Residual Plasma HIV-1 RNA

The univariable linear regression analysis indicated a significant direct association between residual plasma HIV-1 RNA and sCD27 among the 102 subjects with HIV-1 RNA ≤11 copies/mL (Table 3). There was a weaker direct association with sCD30, whereas no association was evident with other markers of immune activation. There was also no association between residual plasma HIV-1 RNA and total HIV-1 DNA and 2-LTRc DNA. The direct association between residual plasma HIV-1 RNA and sCD27 persisted after adjusting for the duration of suppressive ART, frequency of CD8^+^CD38^+^ cells, and levels of sCD30 and IL-6 (Table 3). There was no apparent association between residual plasma HIV-1 RNA and nadir CD4 cell count and pre-ART viral load in the univariable analysis. Forcing the nadir CD4 cell count and pre-ART viral load into the multivariable model did not change the findings: residual plasma HIV-1 RNA was 0.44 log_10_ copies/mL higher per each log_10_ increase in sCD27 (95% CI, 0.09–0.79; *P* = .02; not shown). The association similarly persisted in analyses including the 2 outliers (not shown).

**Table 3.** Univariable and Multivariable Linear Regression Analysis of Factors Associated With Mean Differences in Levels of Residual Plasma HIV-1 RNA (n = 102)^a^

	Univariate			Multivariable^c^		
	Mean Difference^b^	95% CI	*P*	Mean Difference	95% CI	*P*
Nadir CD4 count per 100 cell/mm^3^ higher	0.01	–0.06 to 0.05	.58			
Pre-ART viral load per log_10_ cps/mL higher	0.00	–0.08 to 0.08	.99			
Duration of suppressive ART per 1 y longer	–0.01	–0.03 to 0.00	.12	–0.01	–0.02 to 0.01	.39
CD4 count per 100 cells/mm^3^ higher	–0.01	–0.02 to 0.02	.92			
CD4/CD8 ratio per 1 unit higher	0.04	–0.11 to 0.17	.62			
2-LTRc DNA per log_10_ cps/10^6^ PBMC higher	0.05	–0.11 to 0.20	.57			
Total HIV-1 DNA per log_10_ cps/10^6^ PBMC higher	–0.01	–0.09 to 0.08	.87			
CD4^+^CD38^+^ per 50% higher	0.08	–0.14 to 0.30	.47			
CD4^+^CD26^+^ per 50% higher	0.08	–0.10 to 0.27	.37			
CD4^+^CD69^+^ per 50% higher	–0.61	–2.97 to 1.75	.60			
CD8 count per 100 cells/mm^3^ higher	0.02	–0.01 to 0.01	.70			
CD8^+^CD38^+^ per 50% higher	0.32	–0.11 to 0.75	.16	0.26	–0.17 to 0.68	.24
CD8^+^HLA-DR/DP/DQ^+^ per 50% higher	–0.03	–0.21 to 0.15	.76			
sCD14 per log_10_ µg /mL higher	0.26	–0.24 to 0.75	.31			
sCD27 per log_10_ U/mL higher	0.49	0.17 to 0.81	.003	0.37	0.01 to 0.73	.02
sCD30 per log_10_ ng/mL higher	0.21	–0.02 to 0.45	.07	0.02	–0.25 to 0.28	.88
IL6 per log_10_ pg/mL higher	0.13	–0.05 to 0.30	.15	0.12	–0.06 to 0.29	.19

Abbreviations: 2-LTRc DNA, 2-LTR circular HIV-1 DNA; ART, antiretroviral therapy; CI, confidence interval; cps, copies; PBMC, peripheral blood mononuclear cell.

^a^The analysis excluded the 2 outliers with residual plasma HIV-1 RNA >11 copies/mL.

^b^Mean difference in log_10_ copies/mL.

^c^Variables with *P* < .2 in the univariable analysis were included in the multivariable analysis.

### Relationship Between Virologic Parameters and Plasma Drug Concentrations

**Figure 2.**
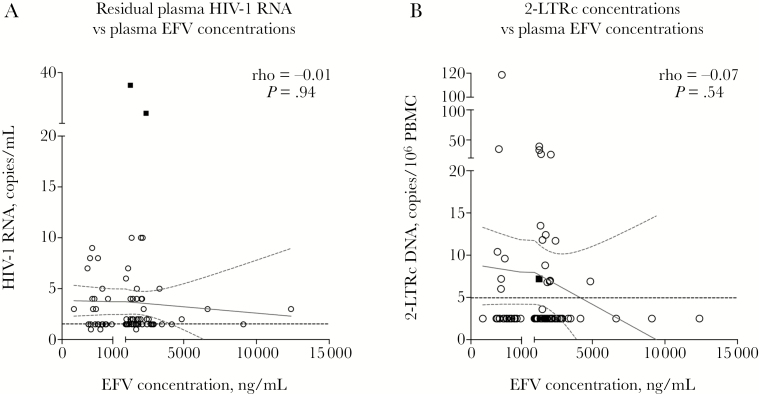
Relationship between efavirenz (EFV) plasma concentrations and (A) residual plasma HIV-1 RNA and (B) 2-LTR circular HIV-1 DNA (2-LTRc DNA) among 76 patients who had taken the last EFV dose a median of 12 hours (interquartile range, 10–13) prior to sampling. Cutoffs in the x-axes represent the minimum effective concentration (MEC) recommended for wild-type virus (1000 ng/mL). The dotted black lines indicate the assay limit of detection (HIV-1 RNA = 3 copies/mL; 2-LTR DNA = 5 copies/10^6^ peripheral blood mononuclear cell). Each circle represents 1 participant; the 2 solid squares represent the 2 patients with residual plasma HIV-1 RNA >11 copies/mL.

We first explored the association between plasma EFV concentrations and virologic parameters in 76 EFV recipients (including the 2 outliers) who had taken the last EFV dose a median (IQR) of 12 (10–13) hours prior to sampling, and were therefore in the range of the “mid-dose” interval, where targets have been previously defined [30]. Although EFV concentrations varied across patients, there was no clear correlation with levels of residual plasma HIV-1 RNA and 2-LTRc DNA (Spearman’s rank test) (Figure 2). Two separate linear regression analyses explored the association between EFV concentrations and residual plasma HIV-1 RNA and 2-LTRc DNA among the 74 patients with residual plasma HIV-1 RNA ≤11 copies/mL. In models that included both EFV concentration and timing of sampling relative to last EFV dose, there was no evidence of an association between plasma HIV-1 RNA (*P* = .30) or 2-LTRc DNA (*P* = .47) and EFV concentrations (Supplementary Table 3).

## DISCUSSION

This study demonstrated that half of subjects who had experienced viral load suppression <50 copies/mL continuously for up to 15 years while maintained on the initial NNRTI had detectable HIV-1 RNA in plasma when tested with an ultrasensitive assay, at levels that rarely exceeded 11 copies/mL. We previously reported that in treated patients, plasma HIV-1 RNA levels above approximately 10 copies/mL were associated with a risk of viral load rebound above 50 copies/mL and above 400 copies/mL over 12 months of follow-up [20]. Taken together, our findings suggest a cutoff level for plasma HIV-1 RNA below which a risk of viral load rebound is not observed. However, an independent, direct association persisted between residual plasma HIV-1 RNA and the immune activation marker sCD27 in this stably treated population.

These data do not elucidate the debated source of residual plasma HIV-1 RNA [15, 31]. The lack of a correlation with levels of 2-LTRc DNA, drug concentrations, or multiple markers of immune activation may indicate that ongoing virus replication was an unlikely source, as far as it can be extrapolated from measurements made in peripheral blood. In this context, the findings may provide support to the notion that during effective treatment, detection of a few HIV-1 RNA copies in plasma reflects transient bursts of virus production from latent reservoirs that are inhibited from ongoing replication by ART [8, 32]. In this model, a direct correlation may be anticipated between the likelihood of HIV-1 RNA persistence and the size of the viral reservoir. We did not detect an association between residual HIV-1 RNA in plasma and total HIV-1 DNA in PBMC, and we similarly did not previously observe an association with integrated HIV-1 DNA in PBMC [21]. Findings from other studies are conflicting, possibly reflecting differences in the composition of the study populations (eg, proportion of patients on protease inhibitors) and the method used for measuring HIV-1 RNA [33]. We also measured 2-LTRc DNA in PBMC as a possible indicator of viral gene expression and virus replication and found no correlation with residual plasma HIV-1 RNA. In a previous study of 30 subjects who had received various ART regimens for 7–12 years, 2-LTRc DNA was detected in 8 subjects (27%) [33], which is highly comparable to our detection rate of 23%. In our study, 2-LTRc DNA levels were lower, with longer duration of suppressive ART, with a reduction of 0.02 log_10_ 2-LTRc DNA copies/10^6^ PBMC per year. This apparent, slow decay in 2-LTRc DNA is consistent with observations from Bushman [34] and Siliciano [35], and potentially in line with the hypothesis that episomal HIV-1 DNA survives for the life of the cell, rather than being a short-lived product of ongoing virus replication [36].

Previous studies did not detect an association between residual plasma HIV-1 RNA and markers of immune activation on CD4 or CD8 cells (including CD38 and HLA-DR) in patients treated for up to 4 years [14, 37], and also detected no association with levels of sCD14 and IL-6 [19]. Our data confirm these observations over the longer term. However, we found that levels of sCD27 were independently associated with levels of residual plasma HIV-1 RNA. sCD27 is a 32-kD protein identical to the extracellular domain of CD27, a transmembrane protein and TNF receptor that is expressed on subsets of T, B, NK, and hematopoietic progenitor cells. sCD27 is released primarily from CD4 T cells and provides a costimulatory function that promotes activation and proliferation of T and B lymphocytes [38–41]. sCD27 can be detected in healthy donors, and increased plasma levels have been observed in conditions characterized by immune activation, especially auto-immune disorders [42–44]. There are limited data in the setting of HIV infection. Levels of sCD27 have been shown to be generally higher than in HIV-negative controls and found to be predictive of the risk of HIV-related renal dysfunction and lymphomas [42, 45]. Levels have been shown to decline significantly in the first 12 to 24 months of ART and to increase again with viral load rebound or upon treatment discontinuation [29, 46, 47]. In our study, consistent with the reported effect of treatment, levels of sCD27 were overall low and reduced further with longer duration of suppressive ART. Taken in the context of these previous observations, our findings illustrate a close association between sCD27 and potential indicators of HIV replication during suppressive ART that warrants further investigation.

There are scarce data on the effect of ART on sCD30 [26]. Levels in our population were considerably lower than those measured in the first 4 weeks of ART [48], and closer to levels published from HIV-negative volunteers [28]; however, sCD30 showed no independent association with virologic parameters. Levels of sCD14, IL-6, and cellular markers of immune activation in our study population were comparable to those previously measured in HIV-1-positive patients receiving ART for up to 6 years [19, 49]. Consistent with previous observations (reviewed in [16]), markers of immune activation on CD4 and CD8 cells (CD38 and HLA-DR/DP/DQ) were lower with longer duration of suppressive ART, and this apparent decline was paralleled by a predictable gain in CD4 cell counts. Overall, as also reported by Gandhi et al. [19], sCD14, IL6, and cellular markers of immune activation showed no apparent association with levels of residual plasma HIV-1 RNA and 2-LTRc DNA.

Our study has limitations. First, findings obtained in a cross-sectional study should be confirmed with prospectively collected data, which is challenging when aiming to study long-term outcomes in populations maintained on the same regimen. To date, prospectively determined virologic indicators of HIV-1 persistence during long-term suppressive ART have been poorly documented. Compared with published studies addressing similar questions, one strength of our study is the relatively large population combined with stringent eligibility criteria and a consistent definition of virologic suppression. It could be argued that our study population was not selected based on the infection stage at the start of ART, which could have introduced a bias. We observed a lower nadir CD4 cell count and a higher pre-ART viral load in subjects with longer duration of suppressive ART; however, the analyses did not identify an effect of nadir CD4 cell count or pre-ART viral load on the measured parameters in our highly selected population. Of note, we excluded 2 outliers from the main analyses with residual plasma HIV-1 RNA levels >11 copies/mL. Whereas the main conclusions were not affected by their inclusion, it would have been of interest to determine any risk of viral load rebound with prospective follow-up in these 2 subjects. Further, we reported total HIV-1 DNA rather than integrated HIV-1 DNA as the indicator of the viral reservoir in this study. However, we previously described integrated HIV-1 DNA levels in a subset of the same population and demonstrated that they were highly correlated with the levels of total HIV-1 DNA, not influenced by the duration of suppressive ART, and not associated with residual HIV-1 RNA [21]. We collected large volumes of blood from patients in order to test for multiple parameters (eg, 8 mL of plasma was required for the ultrasensitive HIV-1 RNA assay alone). Limited sample volumes meant that not all potentially relevant immune parameters were measured. When selecting soluble markers of immune activation, we elected to include 2 markers that have been extensively explored in the published literature (sCD14 and IL-6) to provide a reference and added 2 markers for which there is high plausibility but scarce published evidence (sCD27 and sCD30). In this context, levels of soluble markers of immune activation were found to be similar to those historically reported in HIV-negative volunteers, which is consistent with the established beneficial effects of suppressive ART. However, published data from healthy volunteers were limited, especially for sCD27 and sCD30, and ideally, matched HIV-negative controls should be sampled to confirm reference values. As an additional point, we measured plasma NNRTI concentrations in untimed specimens and for practical reasons did not seek to obtain a measure of C_trough_. The patients analyzed in the models had taken the last EFV dose at a consistent interval relative to sampling, and time of sampling would be expected to have little impact overall. Finally, we recognize that both drug concentration and antiviral activity measured in peripheral blood do not necessarily reflect the situation in lymphoid tissue [8, 9].

In conclusion, this study provides evidence that residual plasma HIV-1 RNA and PBMC-associated 2-LTRc DNA can be detected in a subset of patients receiving long-term, continuously suppressive firstline ART, without evidence of a clear association between the two, and without evidence of an association of either parameter with plasma concentrations of EFV. While residual plasma HIV-1 RNA was not associated with commonly reported markers of immune activation, there was a novel, independent direct association with levels of sCD27. Further studies are warranted to confirm the findings, extend the immune activation profiling, and determine the underlying mechanism, including whether the relationship reflects ongoing virus replication in lymphoid tissue.

## Supplementary Data

Supplementary materials are available at *Open Forum Infectious Diseases* online. Consisting of data provided by the authors to benefit the reader, the posted materials are not copyedited and are the sole responsibility of the authors, so questions or comments should be addressed to the corresponding author.

ofy032_suppl_supplementary_tables

ofy032_suppl_supplementary_figure1

ofy032_suppl_supplementary_figure2

ofy032_suppl_supplementary_figure_legends
