# Supplementary material for: Factors Associated With Persistence of Plasma HIV-1 RNA During Long-term Continuously Suppressive Firstline Antiretroviral Therapy
Source: Open Forum Infect Dis. 2018 Feb 3;5(2):ofy032. doi: 10.1093/ofid/ofy032 (PMC5825920; doi:10.1093/ofid/ofy032)
Supplement: ofy032_suppl_supplementary_tables [file ofy032_suppl_supplementary_tables.docx]

**Supplementary Table 1.** Individual patient data for subjects that showed concomitant elevations of >2 soluble markers of immune activation (n=5)^a^

|  | Patient n | | | | |
| --- | --- | --- | --- | --- | --- |
|  | 01 | 02 | 03 | 04 | 05 |
| Nadir CD4 count, cells/mm^3^ | 218 | 246 | 51 | 267 | 40 |
| Pre-ART HIV-1 RNA, log_10_ cps/ml | 6.0 | 4.7 | 5.7 | 2.8 | 5.3 |
| Duration of suppressive ART, years | 7 | 3 | 8 | 3 | 5 |
| CD4 count, cells/mm^3^ | 507 | 442 | 1068 | 601 | 463 |
| Residual plasma HIV-1 RNA, cps/ml | TND | 7 | TND | 4 | 3 |
| 2-LTRc DNA, cps/10^6^ PBMC | TND | TND | TND | 7 | 10 |
| Total HIV-1 DNA, log_10_ cps/10^6^ PBMC | 2.7 | 3.1 | 2.0 | 2.3 | 1.5 |
| % CD4^+^CD38^+^ | 15 | 31 | 29 | 12 | 19 |
| % CD4^+^CD26^+^ | 57 | 31 | 58 | 29 | 58 |
| % CD4^+^CD69^+^ | 1 | <1 | 2 | 4 | 2 |
| CD8 count, cells/mm^3^ | 706 | 746 | 2753 | 1230 | 2330 |
| % CD8^+^CD38^+^ | 3 | 51 | 3 | 6 | 5 |
| % CD8^+^HLA-DR/DP/DQ^+^ | 39 | 50 | 28 | 77 | 57 |
| sCD14, µg /ml | 2.6 | 2.8 | 2.7 | 1.5 | 3.2 |
| sCD27, U/ml | 85 | 77 | 80 | 114 | 127 |
| sCD30, ng/ml | 19 | 53 | 16 | 25 | 35 |
| IL6, pg/ml | 1.7 | 0.5 | 0.5 | 6.7 | 3.1 |

^a^Shadowed boxes indicate values below (CD4 cell counts) or above (all other parameters) the upper quartile range of the whole study population. ART= Antiretroviral therapy; TND= target non detected; cps= copies; 2-LTRc DNA= 2-LTR circular HIV-1 DNA; PBMC= peripheral blood mononuclear cells.

**Supplementary Table 2.** Univariable and multivariable linear regression analysis of factors associated with mean differences in levels of 2-LTRc DNA (n=102)^a^

|  | Univariate | | | | Multivariable^c^ | | |
| --- | --- | --- | --- | --- | --- | --- | --- |
|  | Mean difference^b^ | 95% CI | P | Mean difference | | 95% CI | P |
| Nadir CD4 count per 100 cell/mm^3^ higher | 0.00 | -0.04, 0.04 | 0.99 |  | |  |  |
| Pre-ART HIV-1 RNA per log_10_ cps/ml higher | 0.06 | -0.03, 0.16 | 0.17 | 0.09 | | -0.01, 0.18 | 0.07 |
| Duration of suppressive ART per 1 year longer | -0.02 | -0.04; 0.00 | 0.09 | -0.03 | | -0.05, -0.00 | 0.02 |
| CD4 count per 100 cells/mm^3^ higher | -0.01 | -0.04, 0.01 | 0.32 |  | |  |  |
| CD4/CD8 ratio per 1 unit higher | -0.04 | -0.22, 0.14 | 0.67 |  | |  |  |
| Residual plasma HIV-1 RNA per log_10_ cps/ml higher | 0.07 | -0.18, 0.33 | 0.56 |  | |  |  |
| Total HIV-1 DNA per log_10_ cps/10^6^ PBMC higher | 0.14 | 0.03, 0.25 | 0.01 |  | |  |  |
| CD4^+^CD38^+^ per 50% higher | 0.05 | -0.23, 0.34 | 0.69 |  | |  |  |
| CD4^+^CD26^+^ per 50% higher | 0.18 | -0.04, 0.42 | 0.11 | 0.15 | | -0.07, 0-38 | 0.18 |
| CD4^+^CD69^+^ per 50% higher | -0.09 | -0.42, 0.23 | 0.58 |  | |  |  |
| CD8 count per 100 cells/mm^3^ higher | -0.01 | -0.02, 0.01 | 0.48 |  | |  |  |
| CD8^+^CD38^+^ per 50% higher | 0.21 | -0.31, 0.73 | 0.43 |  | |  |  |
| CD8^+^HLA-DR/DP/DQ^+^ per 50% higher | 0.01 | -0.24, 0.22 | 0.90 |  | |  |  |
| sCD14 per log_10_ µg /ml higher | 0.26 | -0.37, 0.90 | 0.42 |  | |  |  |
| sCD27 per log_10_ U/ml higher | -0.32 | -0.74, 0.10 | 0.14 | -0.36 | | -0. 77, 0.06 | 0.09 |
| sCD30 per log_10_ ng/ml higher | -0.18 | -0.48, 0.12 | 0.24 |  | |  |  |
| IL6 per log_10_ pg/ml higher | -0.05 | -0.27, 0.18 | 0.68 |  | |  |  |

^a^The analysis excluded the two outliers with residual plasma HIV-1 RNA >11 copies/ml. ^b^Mean difference in log_10_ copies/10^6^ PBMC. ^c^Variables with p<0.2 in the univariable analysis were included in the multivariable analysis, with the exclusion of total HIV-1 DNA. CI= Confidence interval; ART= Antiretroviral therapy; cps= copies; 2-LTRc DNA= 2-LTR circular HIV-1 DNA; PBMC= peripheral blood mononuclear cells.

**Supplementary Table 3**. Multivariable model to test the association between plasma concentrations of EFV and virologic markers in patients with residual HIV-1 RNA ≤11 copies/ml (n=74)^a^

|  | Mean  difference^b^ | 95% CI | P |
| --- | --- | --- | --- |
| Residual plasma HIV-1 RNA log_10_ copies/ml | -0.11 | -0.31; 0.09 | 0.30 |
| 2-LTRc DNA log_10_ copies/10^6^ PBMC | -0.10 | -0.39; 0.18 | 0.47 |

^a^The analysis excluded the two outliers with residual plasma HIV-1 RNA >11 copies/ml. ^b^Mean difference in measured parameters per each log_10_ ng/ml increase in plasma EFV. Both EFV concentration and time of sampling relative to last EFV dose were input into the model.
