# Supplementary figures and images for: Factors Associated With Persistence of Plasma HIV-1 RNA During Long-term Continuously Suppressive Firstline Antiretroviral Therapy
Source: Open Forum Infect Dis. 2018 Feb 3;5(2):ofy032. doi: 10.1093/ofid/ofy032 (PMC5825920; doi:10.1093/ofid/ofy032)

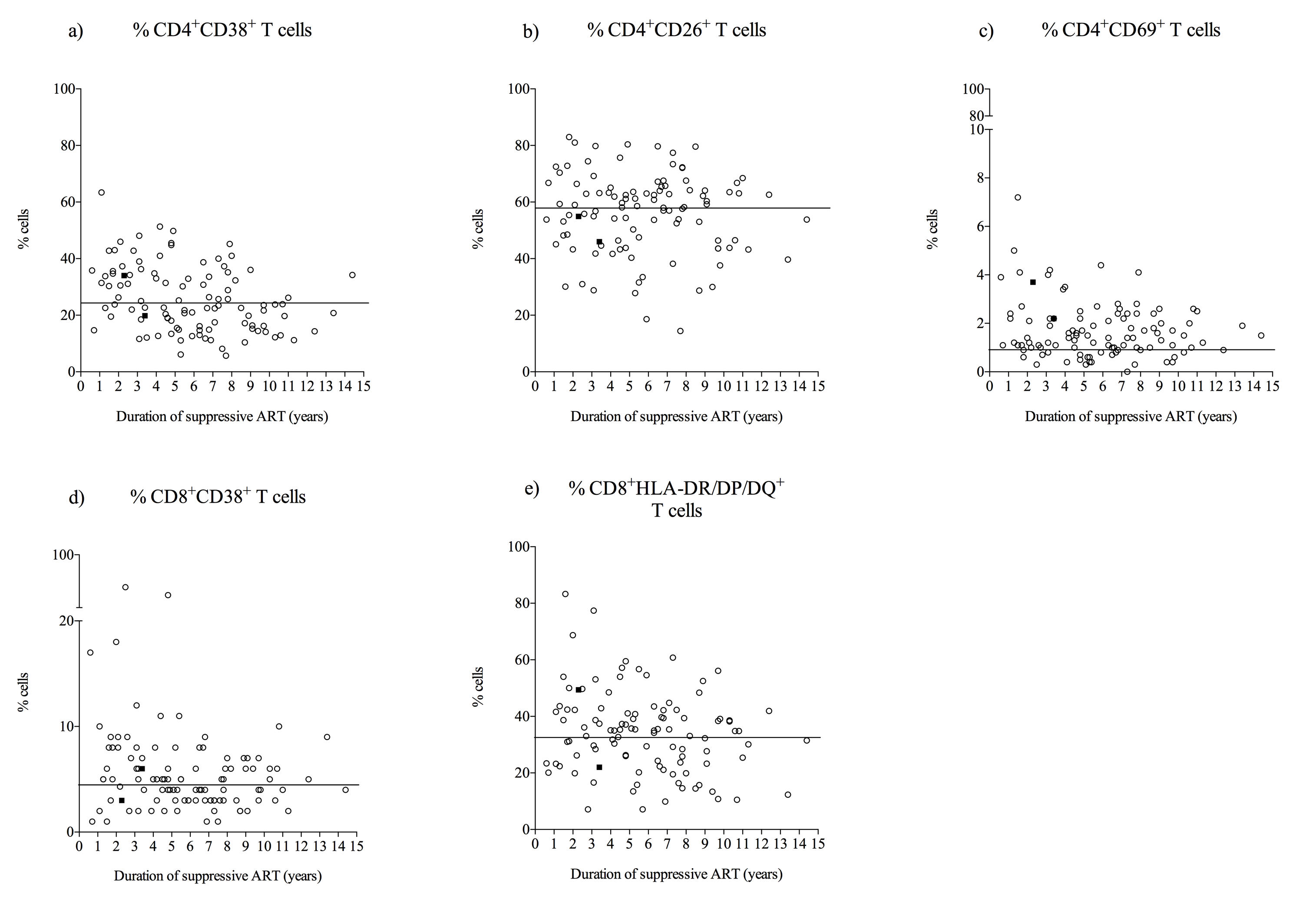

Supplement: ofy032_suppl_supplementary_figure1 [file ofy032_suppl_supplementary_figure1.png]

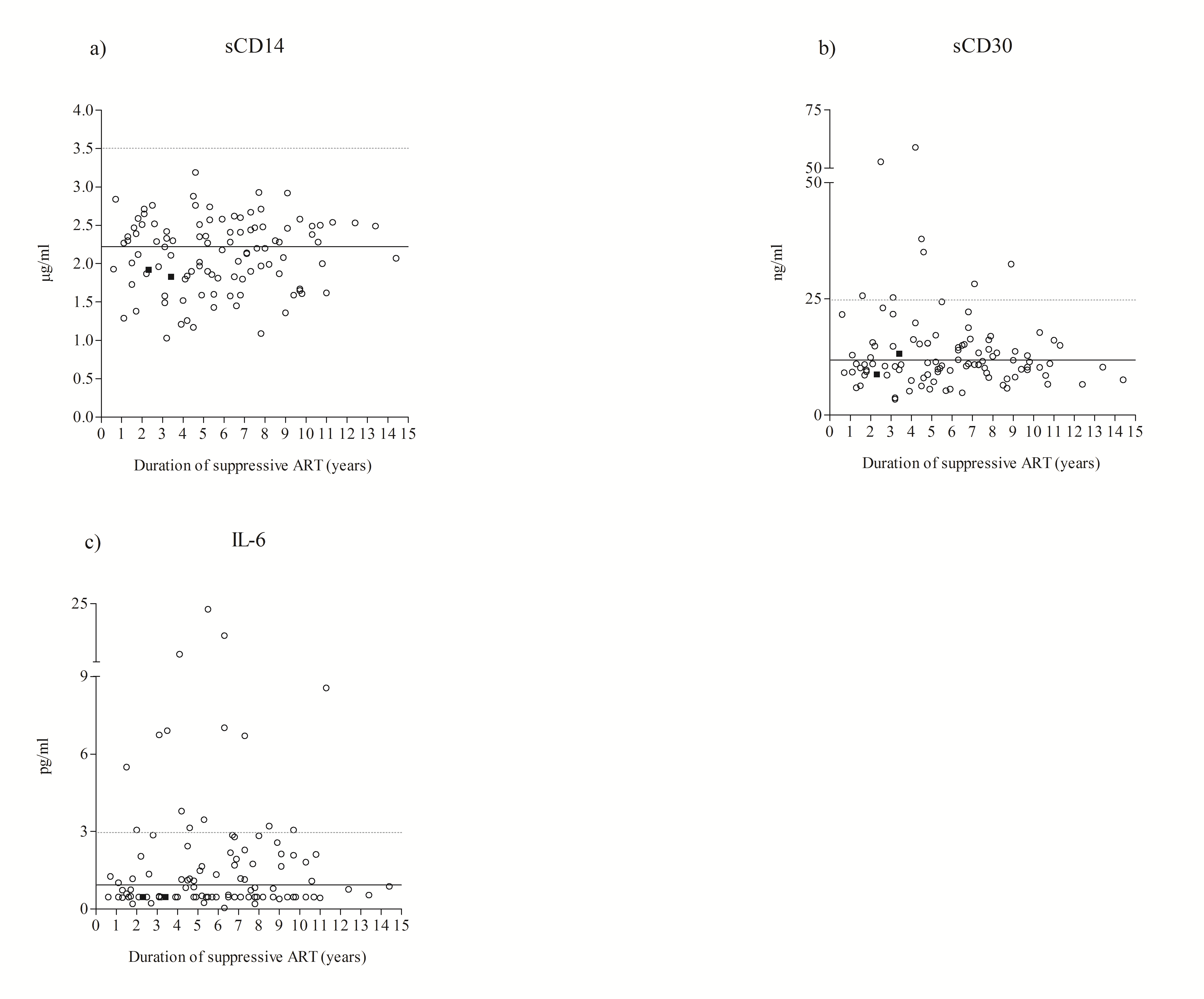

Supplement: ofy032_suppl_supplementary_figure2 [file ofy032_suppl_supplementary_figure2.png]
