# Supplementary material for: Factors Associated With Persistence of Plasma HIV-1 RNA During Long-term Continuously Suppressive Firstline Antiretroviral Therapy
Source: Open Forum Infect Dis. 2018 Feb 3;5(2):ofy032. doi: 10.1093/ofid/ofy032 (PMC5825920; doi:10.1093/ofid/ofy032)
Supplement: ofy032_suppl_supplementary_figure_legends [file ofy032_suppl_supplementary_figure_legends.docx]

**Supplementary figure legends:**

**Supplementary Figure 1.** Cellular markers of immune activation in patients with up to 15 years of continuously suppressive ART (n=104), including: a) CD4^+^CD38^+^; b) CD4^+^CD26^+^; c) CD4^+^CD69^+^; d) CD8^+^CD38^+^; and e) CD8^+^HLA-DR/DP/DQ^+^. The solid lines indicate the median values measured in the whole study population. Each circle represents one participant; the two solid squares represent the two patients with residual plasma HIV-1 RNA >11 copies/ml.

**Supplementary Figure 2.** Soluble markers of immune activation in patients with up to 15 years of continuously suppressive ART (n=104), including: a) sCD14; b) sCD30; and c) IL-6. The dotted lines indicate mean values in HIV-negative volunteers reported in published studies: sCD14 3.5 µg/ml [13]; sCD30 25 ng/mL [25]; and IL-6 2.9 pg/ml [26]. The solid lines indicate the median values measured in the whole study population. Each circle represents one participant; the two solid squares represent the two patients with residual plasma HIV-1 RNA >11 copies/ml.
